# Supplementary material for: Ceftiofur formulation differentially affects the intestinal drug concentration, resistance of fecal Escherichia coli, and the microbiome of steers
Source: PLoS One. 2019 Oct 4;14(10):e0223378. doi: 10.1371/journal.pone.0223378 (PMC6777789; doi:10.1371/journal.pone.0223378)
Supplement: Attachment — Submitted filename: Response to Reviewers.docx [file pone.0223378.s002.docx]

**Thank you to the editors and reviewers for their helpful comments. We have attempted to address all of the stated concerns. The line numbers listed here in our responses correspond to the version of the manuscript without tracked changes.**

Reviewer #2: There are still a lack of information on pharmacokinetics. You need to be more specific about pharmacokinetic parameters as these could affect your outcome. You compared the differences in only plasma concentrations. Why did you not compare the differences in other pharmacokinetic parameters? The Tmax and AUC of CCFA in plasma were difference than CHCL in plasma. Additionally, the Cmax of CCFA in plasma is lower than of CHCL in plasma. The t1/2λz CCFA in plasma was longer than CHCL in plasma. Could you suggest some reasons?

*AU: Our apologies that this was not more clearly stated, but we have compared the PK parameters in all the different matrices and highlighted those in Table 1 that were significantly different. In lines 291-298, we have expanded our description the observed differences in the PK parameters and have added information in lines 387-390 along with a reference highlighting the impact of the slow-release formulation of CCFA.*
